# Women’s perceptions and reasons for choosing the pill, patch, or ring in the CHOICE study: a cross-sectional survey of contraceptive method selection after counseling

**DOI:** 10.1186/1472-6874-13-9

**Published:** 2013-02-28

**Authors:** Christian Egarter, Brigitte Frey Tirri, Johannes Bitzer, Vyacheslav Kaminskyy, Björn J Oddens, Vera Prilepskaya, Arie Yeshaya, Maya Marintcheva-Petrova, Steven Weyers

**Affiliations:** 1 Medical University of Vienna, Vienna, Austria; 2 Frauenklinik, Kantonsspital Baselland, Bruderholz, Switzerland; 3 Frauenklinik, Universitätsspital Basel, Basel, Switzerland; 4 P.L. Shupik National Medical Academy of Post-Graduate Education, Kiev, Ukraine; 5 Global Medical Affairs, Merck, Sharpe & Dohme Corp., Oss, The Netherlands; 6 Scientific Center of Obstetrics, Gynecology and Perinatology of the Ministry of Health of the Russian Federation, Moscow, Russian Federation; 7 Department of Obstetrics and Gynecology, Sackler School of Medicine, Tel Aviv University, Tel Aviv, Israel; 8 Biostatistics and Research Decision Sciences, Merck, Sharpe & Dohme Corp., Oss, The Netherlands; 9 Department of Obstetrics and Gynaecology, Ghent University Hospital, Ghent, Belgium

**Keywords:** Combined oral contraceptive, Contraceptive transdermal patch, Contraceptive vaginal ring, Counseling, Reason, Perception

## Abstract

**Background:**

The European CHOICE study was a cross-sectional survey that evaluated women’s combined hormonal contraceptive choices before and after contraceptive counseling in Austria, Belgium, Czech Republic and Slovakia, the Netherlands, Poland, Sweden, Switzerland, Israel, Russia, and Ukraine. The changes in method selection before and after counseling were reported previously. In this paper we present the reasons given by the 18,787 participating women for selecting their contraceptive method of choice, as well as their perceptions about the contraceptive pill, patch, and ring after counseling.

**Methods:**

Women with an interest in a combined hormonal contraceptive method (pill, patch, or ring) were counseled using a standardized counseling leaflet. The women completed questionnaires, which included questions on why they had selected a particular method of contraception, and the extent to which they agreed with statements about the attributes of the pill, patch, and ring. The results for each country were compared with the percentages for all countries combined by using a binomial regression model. Multiple logistic regression models were used to investigate the extent to which the probability of choosing a method was related to prespecified aspects (i.e. perceptions) of each contraceptive method.

**Results:**

‘Easy to use’, ‘convenience’, and ‘regular menstrual bleeding’ were important selection criteria. ‘Nondaily administration’ was one of the main reasons women selected the patch or ring. ‘Daily use’ and ‘will forget to take it’ were the primary reasons for not selecting the pill, while the main reasons for not choosing the patch included ‘not discrete, visible’ and ‘can fall off’. In a small number of instances, the ring was rejected because some women don’t like to use a ‘foreign body’. Women’s perceptions influenced their contraceptive decisions: positive perceptions about a method increased the likelihood that a woman would select it. After counseling, many women associated the pill with forgetfulness, and many still did not know about the patch or ring’s key attributes. Women’s knowledge about a particular method was generally greater if they had chosen it.

**Conclusions:**

To support informed contraceptive decision-making, healthcare professionals should realize that a woman’s view of a method’s ease of use is more important than perceived efficacy, tolerability, health benefits, or risks.

## Background

Innovation in reversible hormonal contraceptives beyond the pill (e.g. patch, ring, implants, medicated intrauterine system) has given couples more contraceptive choices. Although women in more affluent countries generally have access to a wide range of hormonal contraceptives, combined oral contraceptives (COCs) remain the most popular form of reversible contraception in Europe and the United States [1-3]. Paradoxically, rates of unintended pregnancy and abortion are still remarkably high throughout Europe. Abortion rates (per 1000 women per year) are highest in Sweden (22.5), the United Kingdom (22.4), Estonia (21.0), Romania (19.5), and Hungary (18.0) [3].

Informed health care professional (HCP)–client dialogue and information-sharing may help women select a contraceptive method that best suits their needs and lifestyles, thus maximizing contraceptive compliance and helping to prevent unintended pregnancies [4]. In the Spanish TEAM-06 study, HCPs counseled women about three combined hormonal contraceptive methods (CHCs) that had similar contraceptive efficacy, safety, and tolerability (pill, patch, and ring), but with distinctly different routes of administration (oral, transdermal, and vaginal) and frequency of use (daily, weekly, and monthly) [5].

The Contraceptive Health Research of Informed Choice Experience (CHOICE) study was modeled after the TEAM-06 study and was designed to evaluate women’s contraceptive choices before and after contraceptive counseling in 11 mostly European countries [6]. In the CHOICE study, 47% of women who had an interest in a CHC method selected a method other than the one they originally intended to use after receiving counseling about several CHC methods, including the pill, patch, and ring [6]. The impact of counseling was different between Northern European countries and Central and Eastern European countries, but a significant increase in choosing the patch and ring was observed in all countries.

All women in the CHOICE study received information about three combined hormonal contraceptive methods (pill, patch, and ring). We wanted to understand why they had chosen the method they selected in preference to the alternatives discussed. The women were asked after the counseling to indicate their reasons for selecting their method of choice, including frequency of use, convenience, possibility of forgetting to take it, efficacy, cost, tolerability, safety, familiarity, and non-contraceptive benefits. They were also asked to indicate why they had not chosen an alternative method, for example because of efficacy or tolerability concerns, dislike of a foreign body (ring), lack of knowledge of other users, or their doctor had not recommended an alternative. To assess what women knew about the various methods following counseling, they were asked to comment on the extent to which they agreed, or disagreed, with eight statements about various aspects of the pill, patch, and ring.

The primary CHOICE paper reported the changes in method selection before and after counseling, but did not include the data reported here. In the current paper, we report the reasons and perceptions data for all 11 participating countries. We focus on assessing similarities and differences between the countries, since method selection after counseling varied so much from one country to another. We also present the associations between women’s perceptions about the pill, patch, and ring and the methods women chose. In a third and final paper, we will assess the extent to which the change in method selection (from the pill to another method; or from another method to the patch and ring) was associated with demographic and background characteristics of the women and their healthcare professionals. Although the complete data set about reasons and perceptions, and the associations with the selected method, have not yet been published, some of the data were covered in individual country publications: Belgium (perceptions), the Czech Republic and Slovakia (perceptions), Sweden (reasons and perceptions), and Austria (reasons and perceptions) [7-12].

## Methods

The CHOICE study was a cross-sectional survey that was carried out in eight European Union countries (Austria, Belgium, Czech Republic and Slovakia, the Netherlands, Poland, Sweden, and Switzerland), Israel, the St. Petersburg and Moscow regions of the Russian Federation (Russia), and Ukraine. The study was initiated and financed by the pharmaceutical company MSD. The third author (JB) led a team of representatives of the European Society of Contraception and Reproductive Health (ESC) who: (i) gave expert advice on the design and conduct of the CHOICE study; (ii) added specific questions which were of interest to the ESC to the protocol and the questionnaires; (iii) modified and evaluated the information leaflet about the three contraceptive methods; (iv) developed a comprehensive counseling model as an educational tool to support the study (slides and videos); and (v) guided execution in the countries. MSD invited an expert to join the International Steering Committee from each of the participating countries. The International Steering Committee (listed in the Acknowledgements) discussed and amended the proposed project plan and questionnaires. The Steering Committee designed the counseling leaflet (which was subsequently approved by the ESC) and supervised execution in their country. The questionnaires were modeled after those used in the Spanish TEAM study and the Portuguese IMAGINE study. After input of the ESC and the Steering Committee, the questionnaires were pilot-tested among 5–10 women per country in Belgium, Israel, the Netherlands, Sweden, and Switzerland. This resulted in the addition of a number of answer categories to the questions regarding women’s choice of a particular method.

Three questionnaires were used: a log of all women who consulted the healthcare professional for contraception during the study period, a questionnaire with background characteristics of the healthcare professional, and one-per-subject questionnaire. The third questionnaire was divided into two parts. Part A was completed by the healthcare professional, where he or she indicated which method of contraception the woman contemplated prior to counseling. Part B was completed by the woman and included questions regarding demographic characteristics and gynecological information, which method the woman decided to use after counseling, her reasons for choosing it, her reasons for not choosing other methods, and her perceptions about the pill, patch, and ring. The section on reasons for choosing a particular method contained multiple-choice questions, with 15–17 pre-specified answers and additional space for the women to add their own answers. The perception questions were statements with multiple-choice answers: strongly agree, agree, no opinion, disagree, strongly disagree, and do not know. Part B was designed to take approximately ten minutes to complete.

The details regarding enrollment, selection of women, and statistical considerations are described in detail elsewhere [6]. In brief, women were eligible to participate in the CHOICE study if they were between 15 and 40 years of age, consulted their HCP and expressed an interest in at least one of the CHC methods (pill, patch, or ring), or considered switching from one CHC method to an alternative one. Women who requested a contraceptive method other than the combined pill, patch, or ring and women who excluded one of the three CHC methods were counseled in the usual way, but they did not complete the CHOICE questionnaire and were not considered CHOICE participants. The healthcare professionals (mostly general practitioners in the Netherlands, midwives in Sweden, and gynecologists in all other countries) used a decision flow diagram to identify women who qualified to participate in the study and who were invited to complete the questionnaire. This decision flow diagram has been published elsewhere [6]. The majority of participating women were counseled about the three CHC methods with assistance of a specially designed counseling leaflet that described the advantages and disadvantages of each method [6].

The primary objective of the CHOICE study was to determine the selection rates of the pill, patch, and ring with sufficient precision. The secondary objective was to determine if the selection of a method other than the pill (e.g. patch or ring) undergoes a statistically significant increase after comprehensive contraceptive counseling. The primary and secondary objectives of the CHOICE study led us to choose a target enrollment of 1500 evaluable questionnaires that were to be completed by women from each participating country.

For women who chose a specific method (e.g. pill), the percentage of women who selected this method for a particular reason was compared for each country with the percentage for all countries combined by using a binomial regression model. The same approach was followed for the analysis of reasons for not choosing a method among the group of women who selected one of the other two CHC methods. The models were fit using the GENMOD procedure (with binomial distribution and identity link) in SAS 9.1 (Cary, NC, USA).

We used multiple logistic regression models to investigate the extent to which the probability of choosing a method is related to prespecified aspects (i.e. perceptions) of each contraceptive method. We controlled for country, a woman’s age, other relevant characteristics (e.g. educational level, employment status, plan to have [more] children, unintended pregnancies, steady relationship, last [main] contraceptive method), and characteristics of the HCPs (e.g. gender, age, and most frequently recommended contraceptive method). The data about these potential confounders were taken from the HCP questionnaire and Part B of the per-subject questionnaire.

The predictive factors were selected in a stepwise fashion. Country and age of the woman (continuous variable) were always included in the models. For the stepwise procedure, significance levels of 0.20 and 0.05 were used for inclusion in and exclusion from (respectively) the model. The logistic regression models were fit using the LOGISTIC procedure in SAS 9.1. Women with missing data were excluded.

The adequacy of the final model was assessed by checking the linearity of the estimated logit on the age of women and by the Hosmer-Lemeshow goodness-of-fit test [13]. The ability of the final model to distinguish participants who had chosen each method (pill, patch, or ring) from those who had not chosen a particular method was evaluated by the area under the receiver operating characteristics curve that was captured in the c-statistic. In general, 0.7 ≤ c < 0.8 is considered acceptable discrimination, 0.8 ≤ c < 0.9 is considered excellent discrimination, and c ≥ 0.9 is considered outstanding discrimination [13]. Assuming asymptotic normality, the two-sided 95% Wald confidence intervals (CIs) for the odds ratio (OR) estimates were computed and are presented together with *P*-values.

We expected that women who had a positive opinion about a particular method (e.g. they agreed with its advantages and disagreed with its disadvantages) would have a greater probability of choosing that method relative to the (reference) category of women who said ‘do not know’ or who had ‘no opinion’ about a perception statement (e.g. the [adjusted] OR would be > 1). Conversely, we expected that women who had a negative opinion about a particular method (i.e. they disagreed with its advantages and agreed with its disadvantages) would have a lower probability of choosing that method (OR < 1).

## Results

The CHOICE study (began in April 2009 and ended in October 2010) included 1730 HCPs. The HCP study logs from nine countries (HCPs in Israel and the Netherlands did not keep logs) indicated that 65,603 women consulted their HCPs for contraception during the study period. Of those women, 18,787 expressed an interest in starting or switching to a new CHC method and completed Part B of the study questionnaire. The target number of participants per country was exceeded (range, 1749–2629 women/questionnaires) in all countries except the Netherlands, where only 727 women were recruited. The mean age (± standard deviation) of participating women was 25.8 ± 6.4 years (Table 1).

**Table 1:** Demographic characteristics by country

**Question**	**All**	**Austria**	**Belgium**	**Israel**	**Netherl**	**Sweden**	**Switzerl**	**CZ&SVK**	**Poland**	**Russia**	**Ukraine**
	**(N=18,787)**	**(N=2478)**	**(N=1801)**	**(N=1802)**	**(N=727)**	**(N=1944)**	**(N=2629)**	**(N=1954)**	**(N=1836)**	**(N=1749)**	**(N=1867)**
***Age, mean (SD)***	25.8 (6.4)	24.9 (6.5)	27.8 (6.3)	26.7 (6.0)	24.4 (7.0)	22.6 (6.1)	23.7 (6.3)	26.6 (6.7)	26.8 (5.9)	27.3 (5.7)	27.4 (5.8)
***Highest educational level, n (%)***											
*Primary school*	2163 (11.6)	396 (16.2)	34 (1.9)	51 (2.8)	87 (12.2)	195 (10.1)	1032 (39.9)	241 (12.4)	72 (3.9)	24 (1.4)	31 (1.7)
*Completed high school*	5565 (29.8)	832 (33.9)	581 (32.3)	612 (34.2)	339 (47.7)	1041 (53.9)	710 (27.4)	298 (15.3)	753 (41.2)	180 (10.4)	219 (11.8)
*Advanced education*	5192 (27.8)	728 (29.7)	826 (46.0)	469 (26.2)	225 (31.6)	198 (10.2)	472 (18.2)	1014 (52.0)	239 (13.1)	466 (26.9)	555 (29.8)
*University*	5724 (30.7)	495 (20.2)	355 (18.9)	660 (36.8)	60 (8.4)	498 (25.8)	374 (14.5)	397 (20.4)	762 (41.7)	1065 (61.4)	1058 (56.8)
*Missing*	143	27	5	10	16	12	41	4	10	14	4
***Employment status, n (%)***											
Not employed	5614 (30.5)	846 (35.3)	518 (29.3)	410 (23.0)	169 (24.9)	901 (47.3)	726 (28.4)	592 (31.5)	582 (31.9)	414 (23.9)	456 (24.5)
Part-time employed	3619 (19.7)	452 (18.9)	263 (14.9)	531 (29.7)	316 (46.5)	506 (26.5)	509 (19.9)	184 (9.8)	256 (14.1)	262 (15.1)	340 (18.3)
Full-time employed	9147 (49.8)	1096 (45.8)	988 (55.9)	845 (47.3)	194 (28.6)	499 (26.2)	1318 (51.6)	1103 (58.7)	984 (54.0)	1054 (60.9)	1066 (57.3)
Missing	407	84	32	16	48	38	76	75	14	19	5
**Do you plan to have (more) children later?**											
*No*	3420 (18.5)	351 (14.4)	479 (26.7)	214 (11.9)	110 (15.4)	192 (10.7)	259 (10.1)	503 (26.0)	475 (26.2)	340 (19.6)	497 (26.7)
*Yes*	11,552 (62.6)	1562 (63.9)	1027 (57.3)	1365 (76.2)	410 (57.4)	1077 (60.2)	1802 (70.0)	1172 (60.6)	1028 (56.7)	1130 (65.0)	979 (52.6)
*Do not know yet*	3479 (18.9)	531 (21.7)	286 (16.0)	213 (11.9)	194 (27.2)	521 (29.1%)	512 (19.9)	258 (13.3)	310 (17.1)	268 (15.4)	386 (20.7)
*Missing*	336	34	9	10	13	154	56	21	23	11	5
**Have you had unplanned pregnancies?**											
*No*	14,848 (79.9)	2196 (89.7)	1638 (91.4)	1444 (80.6)	616 (86.5)	1528 (79.2)	2384 (91.6)	1541 (79.1)	1513 (83.1)	1041 (60.2)	947 (52.4)
*Yes*	3732 (20.1)	252 (10.3)	154 (8.6)	348 (19.4)	96 (13.5)	401 (20.8)	220 (8.4)	407 (20.9)	307 (16.9)	687 (39.8)	860 (47.6)
*Missing*	207	30	9	10	15	15	25	6	16	21	60
**Have you had induced abortions?**											
*No*	13,423 (82.2)	2164 (91.9)	1603 (94.2)	1481 (84.2)	633 (90.8)	1576 (82.9)	2357 (92.5)	1623 (84.6)	0	1025 (59.9)	961 (55.1)
*Yes*	2914 (17.8)	191 (8.1)	99 (5.8)	277 (15.8)	64 (9.2)	325 (17.1)	192 (7.5)	296 (15.4)	1 (100)	686 (40.1)	783 (44.9)
*Missing*	2450	123	99	44	30	43	80	35	1835	38	123
**Are you in a steady relationship with a partner?**											
*No*	3181 (17.0)	615 (25.0)	257 (14.3)	264 (14.8)	199 (27.9)	392 (20.3)	467 (17.9)	321 (16.5)	266 (14.5)	165 (9.5)	235 (12.6)
*Yes*	15,492 (83.0)	1843 (75.0)	1541 (85.7)	1522 (85.2)	513 (72.1)	1543 (79.7)	2147 (82.1)	1621 (83.5)	1566 (85.5)	1569 (90.5)	1627 (87.4)
*Missing*	114	20	3	16	15	9	15	12	4	15	5
**Last contraceptive method (four most cited)**											
*Combined oral contraceptive pill*	7652 (41.8)	1179 (49.3)	1206 (67.4)	844 (47.9)	314 (44.7)	828 (43.5)	1245 (48.3)	795 (42.0)	697 (38.7)	175 (10.6)	369 (19.8)
*Condoms*	4622 (25.2)	438 (18.3)	94 (5.3)	379 (21.5)	137 (19.5)	529 (27.8)	608 (23.6)	398 (21.0)	448 (24.9)	783 (47.6)	808 (43.4)
*Natural Family Planning*	1010 (5.5)	49 (2.0)	11 (0.6)	45 (2.6)	5 (0.7)	9 (0.5)	37 (1.4)	100 (5.3)	61 (3.4)	419 (25.5)	274 (14.7)
*I have not used contraception previously*	1877 (10.2)	228 (9.5)	72 (4.0)	144 (8.2)	127 (18.1)	157 (8.3)	254 (9.9)	301 (15.9)	357 (19.8)	112 (6.8)	125 (6.7)

Country: All, all countries combined; Netherl, Netherlands; Switzerl, Switzerland; CZ&SVK, Czech & Slovak Republics.

Many characteristics, including education and full-time employment, varied considerably between countries. Prior to counseling, women were most likely to use COCs (41.8%), condoms (25.2%), natural family planning (5.5%) or no method (10.2%) (Table 1). COC and condom use varied widely between countries. Nearly one-fifth of women had experienced either unintended pregnancy, induced abortion, or both; rates of unintended pregnancy and induced abortion were especially high in Russia (both 40%) and Ukraine (48% and 45%, respectively) and low in Belgium (9% and 6%, respectively) (Table 1) [6].

**Figure 1:**
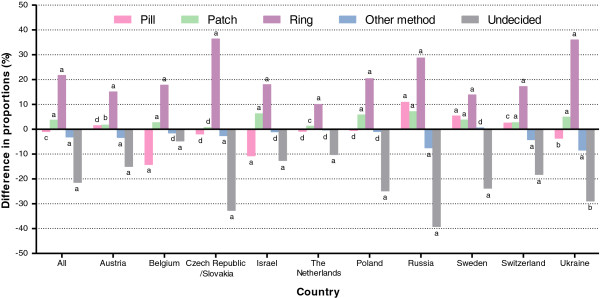
**Difference in proportions between women’s selection of a contraceptive method after counseling versus before counseling (using questionnaires where both post-counseling and pre-counseling contraceptive choices were non-missing).** Pill, combined oral contraceptive pill; patch, transdermal patch; ring, vaginal ring; other method, other contraceptive method; undecided, women did not show a preference for the pill, patch, ring, or other method. ^a^*P* < 0.001; ^b^0.001 *≤ P* < 0.01; ^c^0.01 *≤ P* < 0.05; ^d^not significant (*P* ≥ 0.05).

In the 11 countries combined, a statistically significant increase was noted in the proportion of women who chose the method after counseling versus those who intended to use it before counseling for the patch (+3.7%, 97.5% CI 3.3 to 4.2; *P* < 0.001; McNemar’s test) and the ring (+21.7%, 97.5% CI 21.0 to 22.5; *P* < 0.001) (Figure 1). A statistically significant decrease in proportions was observed for the pill (−0.9%, 95% CI −1.7 to −0.2; *P* = 0.018), other combined methods (−3.1%, 95% CI −3.6 to −2.7; *P* < 0.001), and for women who were undecided regarding which method they preferred to use (−21.4%, 95% CI −22.1 to −20.7; *P* < 0.001). The difference in proportions for each individual country was also calculated.

Reasons for choosing a method after counseling are shown in Table 2 and Additional file 1: Table S1. Women who chose the pill or patch after counseling were most likely to cite ‘easy to use’ as a reason that led to their selection. ‘Regular menstrual bleeding’ and ‘relief from menstrual pain’ were also often cited as a reason why women chose the pill. ‘Nondaily administration’ was the first and second most frequently given reason for selecting the ring and patch, respectively. ‘Will not forget it’ was cited by 56.8% of women who chose the ring and 53.1% of women who chose the patch compared with 21.7% of pill adopters. More than half (60.5%) of women who chose the ring did so because their doctor recommended it.

**Table 2:** Reasons women selected the daily pill, weekly patch, or monthly ring after counseling (all countries combined)

**Reasons women selected a particular method, n (%)**	**Pill** **(*n*= 9418)**	**Patch** **(*n*= 1541)**	**Ring** **(*n*= 5520)**
Daily use	3491 (37.1)	—	—
Weekly use	—	1053 (68.3)	—
Monthly use	—	—	4128 (74.8)
Will not forget it	2044 (21.7)	819 (53.1)	3135 (56.8)
Convenience	4197 (44.6)	1039 (67.4)	3361 (60.9)
Easy to use	6351 (67.4)	1165 (75.6)	3232 (58.6)
My friend uses it	2550 (27.1)	315 (20.4)	1026 (18.6)
I am used to it	3358 (35.7)	80 (5.2)	211 (3.8)
Discrete	2365 (25.1)	—	1815 (32.9)
Can check it, visible	—	632 (41.0)	—
Recommended by my doctor	3140 (33.3)	581 (37.7)	3338 (60.5)
Low hormone levels	2024 (21.5)	498 (32.3)	—
Steady, low hormone levels	—	—	3246 (58.8)
Well-researched method	3720 (39.5)	—	—
Still effective if I experience vomiting	—	671 (43.5)	2721 (49.3)
Still effective with certain antibiotics	—	—	1776 (32.2)
Regular menstrual bleeding	6184 (65.7)	694 (45.0)	2719 (49.3)
Low chance of side effects	1881 (20.0)	570 (37.0)	2665 (48.3)
Not dangerous	1748 (18.6)	497 (32.3)	1859 (33.7)
Relief from menstrual pain	4556 (48.4)	343 (22.3)	1383 (25.1)
Relief from acne	2812 (29.9)	122 (7.9)	468 (8.5)
Other	410 (4.4)	39 (2.5)	141 (2.6)

Patch = transdermal patch; pill = combined oral daily pill; ring = vaginal ring; ‘—’ = not asked for particular method; *n* = number of subjects who selected at least one of the pre-specified reasons; other = other reason not previously specified. Missing responses (number of subjects who did not select any of the pre-specified reasons): daily pill, n = 26; weekly patch, n = 8; monthly ring, n = 20.

Reasons for choosing a method were similar in most countries. However, within certain countries, the most prominent reasons varied from the percentage in all countries combined by a statistically significant margin (*P* < 0.001 for each reason listed in this paragraph). In Ukraine, women who selected the pill were less likely to state ‘daily use’ as one of the reasons they selected the pill. Women in Russia and Ukraine who selected the pill were more likely to state they chose it because it was ‘recommended by my doctor’ and was a ‘well-researched method’. If they chose the patch or ring, women in Russia and Ukraine were more likely to state that they selected these methods because they were ‘not dangerous’. Conversely, women in Belgium and the Netherlands who selected the pill were less likely to cite ‘well-researched method’ as a reason for choosing it. In Austria, ‘convenience’ mattered to fewer women who selected the pill, patch, or ring. ‘Convenience’ also mattered to fewer women in Switzerland who selected the patch and ring. In the Czech Republic and Slovakia, women were more likely to cite ‘weekly use’ and ‘monthly use’ as reasons to choose the patch and ring, respectively. In the Netherlands, women were less likely to state they selected the patch and ring because they are ‘easy to use’. In Poland, ‘discretion’ was more frequently given as a reason for women choosing the pill and ring relative to the percentage in all 11 countries combined.

Reasons for not choosing a method are shown in Table 3 and Additional file 2: Table S2. The top two reasons women did not select the pill included ‘daily use’ and ‘will forget to take it’. Women who did not select the patch cited ‘not discrete, visible’ and ‘can fall off’. For women who did not select the ring, ‘don’t like to use foreign body’ and ‘more convenient methods are available’ were the most common reasons. For 21.8% of women who did not choose the ring, cost was a factor that led them to choose an alternative method.

**Table 3:** Reasons not to choose the pill, patch, or ring (all countries combined)

**Reasons why women who selected the:**	**Patch or ring**	**Pill or ring**	**Pill or patch**
**Did not select the:**	**Pill**^**a**^	**Patch**^**b**^	**Ring**^**c**^
	**(%)**	**(%)**	**(%)**
Daily use	5083 (72.0)	—	—
Not interested in weekly contraception	—	3879 (26.0)	—
Not interested in monthly contraception	—	—	2174 (19.8)
More convenient methods are available	3364 (47.6)	5893 (39.5)	4406 (40.2)
Not easy to use	541 (7.7)	1163 (7.8)	2766 (25.2)
Heard negative stories	1337 (18.9)	1908 (12.8)	993 (9.1)
Not effective	144 (2.0)	411 (2.8)	159 (1.5)
Will forget to take it	4788 (67.8)	—	—
Will forget to remove and replace	—	2877 (19.3)	—
Will forget to remove	—	—	1959 (17.9)
Do not like to detach from skin	—	3851 (25.8)	—
Do not like to use a foreign body	—	—	5308 (48.4)
No regular menstrual bleeding	339 (4.8)	486 (3.3)	436 (4.0)
Very old method	742 (10.5)	—	—
Cost	187 (2.6)	1957 (13.1)	2387 (21.8)
Not effective with certain antibiotics	1958 (27.7)	—	—
Efficacy reduced by vomiting, diarrhea	3167 (44.9)	—	—
Not discrete, visible	—	7911 (53.0)	—
Can fall off	—	7228 (48.4)	—
Can fall out	—	—	2116 (19.3)
Can irritate skin	—	5817 (38.9)	—
Do not know anybody who uses it	63 (0.9)	3920 (26.2)	3709 (33.8)
Doctor did not recommend it	441 (6.2)	1216 (8.1)	653 (6.0)
My partner does not like it	170 (2.4)	595 (4.0)	885 (8.1)
Side effects	2433 (34.5)	481 (3.2)	277 (2.5)
Dangerous	324 (4.6)	75 (0.5)	105 (1.0)
Other	318 (4.5)	727 (4.9)	592 (5.4)

‘—’ = reason not asked for particular method; other = other reason not previously specified.

^a^missing responses, n = 28; ^b^missing responses, n = 47; ^c^missing responses, n = 34.

Within certain countries, many of the reasons that women cited for not choosing the pill, patch, or ring varied from the (pooled) response across all countries combined by a statistically significant margin (*P* < 0.001 for each reason listed in this paragraph). In Belgium, Israel, and the Netherlands, women were less likely to cite ‘daily use’ as a reason for not selecting the pill. Women in Austria, Belgium, Israel, and the Netherlands were less likely to cite ‘more convenient methods are available’ as the reason they did not select the pill. In Poland, women who did not select the ring were more likely to state they were ‘not interested in monthly contraception’. In Russia, women who did not select the patch were more likely to cite that the patch ‘can irritate skin’. Women in the Netherlands were more likely to avoid selecting the patch because they did not know anybody else who used it. Women in the Czech Republic/Slovakia and Ukraine were more likely to cite cost as a reason for not choosing the ring. In Russia and Ukraine, women were more likely to state that they ‘do not like to use a foreign body’ as a reason for not choosing the ring. In contrast, women in Belgium, Sweden, and Switzerland were less likely to cite ‘do not like to use a foreign body’ as the reason for rejecting the ring. In Ukraine, women more often stated that their partner did not like the ring.

In Table 4, women’s perceptions about the pill, patch, or ring after counseling are shown for all countries combined. While more than 90% of women strongly agreed that the pill ‘prevents pregnancy effectively’, fewer than 75% of women strongly agreed that the patch and ring do so. Although a lot more women strongly agreed that the pill had ‘many side effects’ than the patch or ring, approximately the same percentage of women strongly disagreed with this statement. About one quarter of women strongly agreed that the pill ‘can be dangerous for your health’, yet fewer than 10% of women had the same belief about the patch and ring. More women believed the pill was ‘easy to use’ and ‘easy to forget’ compared with the patch or ring. Women were also more likely to associate ‘regular menstrual bleeding’ with the pill than the patch or ring. More than 85% of women strongly agreed that many women use the pill, although only 15% had the same perceptions about the patch or ring.

**Table 4:** **Women’s perceptions about the pill, patch, and ring after counseling**^**a**^

**Perception statement**	**Method**	**Strongly agree/agree**	**No opinion**	**Strongly disagree/disagree**	**Do not know**	**Missing**
		**n (%)**	**n (%)**	**n (%)**	**n (%)**	**(n)**
Prevents pregnancy effectively	Pill	17232 (93.0)	680 (3.7)	314 (1.7)	309 (1.7)	252
	Patch	11809 (65.4)	2941 (16.3)	480 (2.7)	2826 (15.7)	731
	Ring	13185 (73.1)	2189 (12.1)	283 (1.6)	2377 (13.2)	753
Many side effects	Pill	6609 (35.7)	4699 (25.4)	6285 (34.0)	894 (4.8)	300
	Patch	2580 (14.4)	6500 (36.2)	4084 (22.7)	4806 (26.7)	817
	Ring	1696 (9.4)	5697 (31.7)	6061 (33.7)	4523 (25.2)	810
Can be dangerous for your health	Pill	4704 (25.5)	4837 (26.2)	7620 (41.3)	1281 (6.9)	345
	Patch	1694 (9.5)	5086 (28.4)	6886 (38.4)	4248 (23.7)	873
	Ring	1295 (7.2)	4637 (25.8)	8100 (45.1)	3922 (21.8)	833
Easy to use	Pill	14920 (81.0)	1605 (8.7)	1687 (9.2)	218 (1.2)	357
	Patch	10566 (59.0)	3199 (17.9)	1813 (10.1)	2334 (13.0)	875
	Ring	8445 (47.1)	3615 (20.1)	2717 (15.1)	3168 (17.7)	842
Easy to forget	Pill	12034 (65.4)	2625 (14.3)	3440 (18.7)	304 (1.7)	384
	Patch	4945 (27.6)	4712 (26.3)	5495 (30.7)	2766 (15.4)	869
	Ring	3245 (18.2)	4428 (24.8)	7251 (40.6)	2949 (16.5)	914
Regular menstrual bleeding	Pill	15845 (85.9)	1238 (6.7)	626 (3.4)	742 (4.0)	336
	Patch	9240 (51.6)	3618 (20.2)	462 (2.6)	4581 (25.6)	886
	Ring	10067 (56.2)	3185 (17.8)	432 (2.4)	4228 (23.6)	875
Protects against cancer	Pill	5373 (29.2)	4789 (26.0)	3037 (16.5)	5228 (28.4)	360
	Patch	3093 (17.3)	4956 (27.7)	2662 (14.9)	7213 (40.2)	863
	Ring	3377 (18.8)	4772 (26.6)	2541 (14.2)	7252 (40.4)	845
Many women use it	Pill	15816 (85.4)	1371 (7.4)	338 (1.8)	1003 (5.4)	259
	Patch	2157 (12.0)	4757 (26.4)	4339 (24.1)	6737 (37.4)	797
	Ring	2626 (14.6)	4713 (26.2)	3462 (19.3)	7180 (39.9)	806

^a^Percentage of women who strongly agreed/agreed with statement, strongly disagreed/disagreed with statement, and ‘had no opinion’ or ‘did not know’ about statement related to the pill, patch, or ring. Data include all women (i.e. data not restricted to women who selected or did not select a particular method).

We also looked at these perceptions in the subset of women who eventually selected the method concerned (Table 5). Although more women in the total sample agreed that the pill is effective compared with those who thought the patch or ring are effective (Table 4), the percentage who agreed that their selected method is effective were similar for all three methods. Women who selected the pill after counseling were more likely to believe that it had ‘many side effects’ (25.2%) compared with women who had chosen the patch (10.1%) or ring (6.7%). Although 18.3% of women believed that the pill ‘can be dangerous for your health’, one in three women who selected the pill ‘did not know’ or had ‘no opinion’ regarding its dangers. Nearly all women who chose the pill, patch, or ring felt that their method was ‘easy to use’. More than half of women who chose the pill agreed that it is ‘easy to forget’; this is in contrast to the 15.3% of women who chose the patch and 13.3% who chose the ring. Women believed that all three chosen methods provided good cycle control. More than 50% of women who chose the pill, patch, or ring ‘did not know’ or had ‘no opinion’ regarding whether the method they chose protected against some types of cancer.

**Table 5:** **Women’s perceptions about the pill, patch and ring after counseling**^**a**^

		**Women who selected specified method**					**Women who did not select specified method**			
**Perception statement**	**Method**	**Strongly agree/agree n (%)**	**Strongly disagree/disagree n (%)**	**No opinion/do not know n (%)**	**Missing (n)**	**Method (%)**	**Strongly agree/agree (%)**	**Strongly disagree/disagree (%)**	**No opinion/do not know (%)**	**Missing (n)**
Prevents pregnancy effectively	Pill	8969 (95.7)	92 (1.0)	309 (3.3)	74	Pill	8263 (90.2)	222 (2.4)	680 (7.4)	178
	Patch	1284 (84.7)	11 (0.7)	221(14.6)	33	Patch	10525(63.6)	469 (2.8)	5546(33.5)	698
	Ring	4837 (89.1)	13 (0.2)	580 (10.7)	110	Ring	8348 (66.2)	270 (2.1)	3986(31.6)	643
Many side effects	Pill	2351 (25.2)	3941 (42.3)	3029 (32.5)	123	Pill	4258 (46.5)	2344 (25.6)	2564 (28.0)	177
	Patch	151 (10.1)	649 (43.4)	696 (46.5)	53	Patch	2429 (14.7)	3435 (20.9)	10610 (64.4)	764
	Ring	361 (6.7)	2861 (52.9)	2182 (40.4)	136	Ring	1335 (10.6)	3200 (25.5)	8038 (63.9)	674
Can be dangerous for your health	Pill	1704 (18.3)	4528 (48.7)	3073 (33.0)	139	Pill	3000 (32.8)	3092 (33.8)	3045 (33.3)	206
	Patch	119 (7.9)	804 (53.7)	574 (38.3)	52	Patch	1575 (9.6)	6082 (37.0)	8760 (53.4)	821
	Ring	339 (6.3)	3276 (60.7)	1785 (33.1)	140	Ring	956 (7.6)	4824 (38.4)	6774 (54.0)	693
Easy to use	Pill	8627 (92.5)	214 (2.3)	489 (5.2)	114	Pill	6293 (69.2)	1473 (16.2)	1334 (14.7)	243
	Patch	1318 (87.7)	34 (2.3)	151 (10.1)	46	Patch	9248 (56.4)	1779 (10.8)	5382 (32.8)	829
	Ring	4188 (77.5)	149 (2.8)	1067 (19.7)	136	Ring	4257 (33.9)	2568 (20.5)	5716 (45.6)	706
Easy to forget	Pill	4894 (52.7)	2457 (26.5)	1931 (20.8)	162	Pill	7140 (78.3)	983 (10.8)	998 (10.9)	222
	Patch	229 (15.3)	860 (57.6)	405 (27.1)	55	Patch	4716 (28.7)	4635 (28.2)	7073 (43.1)	814
	Ring	713 (13.3)	3179 (59.2)	1475 (27.5)	173	Ring	2532 (20.2)	4072 (32.6)	5902 (47.2)	741
Regular menstrual bleeding	Pill	8274 (88.8)	221 (2.4)	825 (8.9)	124	Pill	7571 (82.9)	405 (4.4)	1155 (12.7)	212
	Patch	1063 (70.7)	31 (2.1)	410 (27.3)	45	Patch	8177 (49.9)	431 (2.6)	7789 (47.5)	841
	Ring	4064 (75.5)	86 (1.6)	1233 (22.9)	157	Ring	6003 (47.9)	346 (2.8)	6180 (49.3)	718
Protects against cancer	Pill	2686 (28.9)	1542 (16.6)	5076 (54.6)	140	Pill	2687 (29.5)	1495 (16.4)	4941 (54.2)	220
	Patch	358 (23.9)	204 (13.6)	938 (62.5)	49	Patch	2735 (16.7)	2458 (15.0)	11231 (68.4)	814
	Ring	1457 (27.0)	645 (11.9)	3299 (61.1)	139	Ring	1920 (15.3)	1896 (15.1)	8725 (69.6)	706
Many women use it	Pill	8361 (89.3)	82 (0.9)	920 (9.8)	81	Pill	7455 (81.3)	256 (2.8)	1454 (15.8)	178
	Patch	413 (27.4)	185 (12.3)	910 (60.3)	41	Patch	1744 (10.6)	4154 (25.2)	10584 (64.2)	756
	Ring	1316 (24.4)	706 (13.1)	3379 (62.6)	139	Ring	1310 (10.4)	2756 (21.9)	8514 (67.7)	667

^a^Percentage of women who strongly agreed/agreed with statement, had ‘no opinion’ of statement, strongly disagreed/disagreed with statement, or ‘did not know’ about statement related to the pill, patch, or ring. Women’s perceptions are included for those who chose and did not choose the stated method. Women who did not choose the stated method could have chosen another method or were undecided. Data in this table are restricted to women who chose a particular method and those who chose a method other than the listed method.

We also looked at women’s perceptions among those who did not choose the method concerned (Table 5). This analysis indicated that women most often agreed or strongly agreed with the statements about the pill (e.g. the method is effective, has ‘many side effects’, is ‘easy to use’, is ‘easy to forget’, provides regular cycle control, and is used by many women). While women who did not choose the patch or ring recognized that the patch and ring are effective, they often ‘did not know’ or had ‘no opinion’ about the other statements.

**Figure 2:**
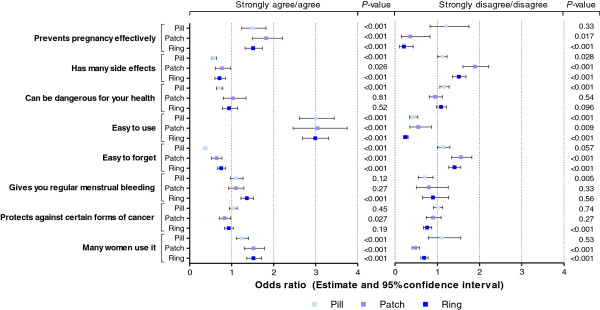
**Results of the multiple logistic regression analyses relating to a woman’s likelihood of choosing the pill, patch, or ring to the perception statements about these methods.** The models fit the data well (c-statistic was 0.795, 0.802, and 0.820 for the models for the pill, patch, and ring, respectively, which was considered acceptable to excellent discrimination). The likelihood of selecting the pill, patch, or ring is higher if the odds ratio (OR) is > 1 or lower if the OR is < 1 for women who ‘strongly agree/agree’ (or ‘strongly disagree/disagree’) compared with women who indicated they had ‘no opinion’ or ‘do not know’ about the perception statement in relation to the method. Two-sided 95% confidence intervals (CIs) are based on asymptotic normality of the OR estimates. The likelihood of selecting a method is higher (by a statistically significant margin at 5% level) if the lower limit of the CI is > 1. Conversely, the likelihood of selecting a method is lower (by a statistically significant margin at 5%) if the upper limit of the CI is < 1. If the CI contains 1, women who ‘strongly agree/agree’ (or ‘strongly disagree/disagree’) compared with those who had ‘no opinion’ or ‘do not know’ do not differ by a statistically significant margin with respect to the probability of choosing the method.

In Figure 2, results are shown from the multiple logistic regression models investigating the extent to which the probability of choosing a particular method (pill, patch, or ring) was related to women’s perceptions about these methods. Women who agreed or strongly agreed that the pill is effective, ‘easy to use’, and that ‘many women use it’ had a higher probability (statistically significant at the 5% level) of choosing this method compared with women who had ‘no opinion’ or ‘do not know’ (the reference category). Women who disagreed or strongly disagreed that the pill has ‘many side effects’ and ‘can be dangerous for your health’ had a higher probability of choosing it compared with the reference category. Women who agreed that the patch is effective, ‘easy to use’, and that ‘many women use it’ or disagreed that it has ‘many side effects’ and is ‘easy to forget’ had a significantly higher probability of choosing it compared with women who had ‘no opinion’ or ‘do not know’. Women who agreed that the ring is effective, ‘easy to use’, allows ‘regular menstrual bleeding’, and that ‘many women use it’ or disagreed that it has ‘many side effects’ and is ‘easy to forget’ had a significantly higher probability of choosing the ring compared with women who had ‘no opinion’ or ‘do not know’. For the pill, patch, and ring, ‘easy to use’ was the strongest distinguishing factor between adopters and non-adopters.

Agreement or disagreement with the statements the patch ‘can be dangerous for your health’ and enables ‘regular menstrual bleeding’ did not appear to relate to women’s selection of the patch. In addition, agreement or disagreement that the ring ‘can be dangerous for your health’ had no relationship with selection of the ring. A woman's perception of the statement ‘protects against certain forms of cancer’ did not typically influence whether a woman chose a method, yet women who disagreed with this statement for the ring were less likely to choose it.

## Discussion

In this study, nearly half of women who underwent structured contraceptive counseling selected a CHC method that was different from the method they originally intended to use [6]. After informed dialogue, one in four women who intended to use the pill selected another method (16.4% and 65.2% of these women chose the patch and ring, respectively) [6]. Despite these important changes, women throughout Europe still prefer the pill, the method with which they are most familiar. This finding has been observed in previous reports [1,14].

Although there are many reasons why women choose one form of contraception over another, we tried to determine the most common reasons that may have influenced women’s choices. Throughout Europe, ‘easy to use’ is an important reason for choosing the pill and patch. ‘Nondaily administration’ is important for women’s selection of the patch and ring. Matters of convenience seem to be especially important for women’s selection of the patch or ring. Women who did not choose the pill did so because of its daily use, the likelihood of forgetting it, ‘more convenient methods are available’, and because the efficacy of the pill is reduced by vomiting or diarrhea. The patch was often not selected because it is ‘not discrete, visible’ and ‘can fall off’. The ring was often not selected because women ‘do not like to use a foreign body’ and ‘more convenient methods are available’.

Knowledge of contraceptive methods obtained as a result of counseling appeared to be a major relevant factor. Women’s perceptions varied for each method, yet women who did not select a specified method were more likely to answer ‘do not know’ to a given statement than women who selected a particular method. If a woman is less knowledgeable about a method, her HCP can help her become better informed. This observation reinforces the need for HCPs to provide comprehensive contraceptive counseling that encourages an exchange of information between HCPs and women. For all listed reasons, the percentage of women who agreed with statements given to them was consistently high for the pill.

In Central and Eastern Europe, more women switched to the patch or ring after counseling. In these regions, we speculate that counseling made them overcome an initial lack of knowledge about alternatives to the pill; they often cited ‘recommended by my doctor’ as a reason for choosing the patch or ring. Indeed, other reports already indicated that HCP preferences had a greater influence on women’s contraceptive choices in Central and Eastern European countries compared with Northern European countries [6].

In Belgium and the Netherlands, women were less likely to cite ‘well-researched method’ as the reason they selected the pill, possibly because they believe the patch and ring are also well-researched methods. Safety concerns seemed to matter less in Belgium and the Netherlands, but played more of a prominent role in Russia and Ukraine.

The difference in the percentage of women who agreed that the pill, patch, or ring prevents pregnancy effectively may have been influenced by the relatively large number of women in the total study sample who selected the pill. Therefore, we specifically looked at perceptions of women who selected the method concerned. The percentages of women who agreed that their chosen method was effective in preventing pregnancy, easy to use, and effective in cycle regulation appeared to be similar for each method. Surprisingly, more women who chose the pill than the patch or ring thought the method had side effects and could be dangerous. Protection against certain forms of cancer was not recognized in any of the three methods. In a study that assessed the perceptions and attitudes of Swedish teenage girls (≤ 17 years old), many stated that hormonal contraceptives may cause negative side effects and damage to the body, especially during puberty [15]. The current data indicate that even women who choose the pill may have negative perceptions about their method; these women may have chosen the pill because of a lack of knowledge about alternative methods.

In a recent study that surveyed women in France, Germany, Italy, Spain, and the United Kingdom, social and cultural differences between these countries influenced women’s decisions regarding their method of contraception [1]. Among the selected countries in the CHOICE study, religion, socioeconomic status, and other factors may have influenced women’s contraceptive choices. In the Czech Republic and Slovakia, Poland, and Ukraine, we noted that women placed more emphasis on using discrete forms of contraception, including the pill and ring.

Although ‘easy to use’ and administration frequency were rated highly as selection criteria for the pill, patch, or ring in all countries, ‘convenience’ was cited less often by Austrian and Swiss women who selected the patch or ring compared with women in Poland, Russia, and Ukraine. We speculate that longstanding use of the pill may have contributed to a more negative image regarding the pill’s health risks (e.g. pill scares, media reports), which could have prompted women (even in countries where convenience is less important) to seek alternative contraceptive methods.

A recent study assessed women’s perceptions about the safety of COCs [16]. Of 794 women who were at risk of unintended pregnancy, 56.0% stated that COCs were medically safe, 39.0% believed that COCs were unsafe, and 13.2% were not sure. The top reasons women thought COCs were unsafe included concerns about side effects (19.5%) and prior negative experiences with COCs (17.0%). In the CHOICE study, women who selected the patch or ring but not the pill were almost twice as likely to indicate that COCs have many side effects compared with women in the previous study [16].

In the CHOICE study, the analyses of the association between women’s choice of method and their perceptions were, among other factors, corrected for women’s age, HCP's most frequently recommended method, women's last (main) contraceptive method, as well as other factors. These factors influenced a woman’s decision-making process. However, we also assume that cost played a decisive role in women’s selection of a CHC method. In most of the 11 participating countries, the patch and ring are indeed considerably more expensive than generic COCs, but in general their price is quite similar to the newer, branded COCs. Surprisingly, only 13% and 22% of participants did not choose the patch or ring, respectively, because of cost. Nevertheless, in the Czech Republic and Slovakia and Russia, cost was a significant deterrent that prevented many women from selecting the ring.

Our analysis of the association between a woman’s selection of the pill, patch, or ring and her perceptions about these methods after counseling indicated that ease of use was the most important knowledge factor. In addition, women who chose a method had a positive impression about the method's efficacy, tolerability, safety, and cycle control. It was disappointing to see that, following counseling, so many women still indicated that they ‘did not know’ or had ‘no opinion’ about the advantages and disadvantages of the patch and ring (unless they had chosen this method). Furthermore, most women had an opinion (positive or negative) about the pill, irrespective of whether they had chosen it.

The CHOICE study had several limitations. First, the study focused on women who were considering a combined hormonal method – women considering other forms of contraception or who indicated prior to the counseling they would not consider one of the three CHC methods did not complete the questionnaires and were counseled in the usual way. This was a deliberate decision, in consultation with the ESC, since we wanted to assess to what extent women consider alternatives to the method they were contemplating, provided it was a viable alternative for them. Nevertheless, this focus limits the generalizability of the findings only to women in the same situation. Secondly, we did not assess contraceptive compliance, discontinuation rates, or user satisfaction. This would have necessitated the need for a follow-up visit, which would have been costly and was not the primary interest of the program. Furthermore, in Belgium, Russia, and Ukraine, HCP’s favorable attitudes towards the patch and ring may have influenced the information and advice they provided, leading to a greater proportion of women who selected the patch or ring [6]. The questionnaire may have had limitations in that women could not find the answer they wished to select. After pilot-testing in a number of countries, we included more answer categories to the questions relating to method of choice over other methods, while leaving space for women to provide their own responses. It was reassuring to see that only 4-5% of participants used this space (summarized as ‘other’ in Tables 1 and 2). Finally, because of slow enrollment, we did not meet our target enrollment of 1500 women in the Netherlands, which diminished the accuracy of the estimated selection rates of the pill, patch, and ring in that country. We attribute low enrollment to the fact that in the Netherlands, general practitioners saw far fewer women per week for contraceptive advice than HCPs in other countries.

The results from the CHOICE study allow us to draw several conclusions. Informed dialogue between HCPs and women encourages women to select alternative contraceptive methods. The majority of women who selected the pill, patch, or ring stated that daily, weekly, or monthly use was an important consideration that factored into their final choice. ‘Daily use’ was the main reason why women who selected the patch or ring did not select the pill. ‘Easy to use’ was the most significant factor affecting women’s choices. Reasons beyond frequency of administration, including visibility of the method (e.g. patch), placement of foreign body inside the body (e.g. ring), and use of a well-known method versus use of a new, more innovative method, were important and varied significantly between countries. Additional knowledge of the various methods following counseling also played an important role. Surprisingly, a woman’s negative feelings about a particular method, especially the pill, did not always dissuade her from selecting it. In Central and Eastern Europe, women’s contraceptive decisions were more driven by safety concerns and HCP recommendations, whereas in Western Europe, women’s decisions were more driven by a less favorable opinion about the pill. While patch and ring use increased in all countries after counseling, women’s reasons for selecting or not selecting a particular method varied considerably between countries.

## Conclusions

In conclusion, we suggest that the results of our study demonstrate that awareness of the decision-making factors that affect women’s choices regarding methods of contraception may enable HCPs to make more informed recommendations that are targeted to the needs of each of their female patients.

## Abbreviations

CHC: Combined hormonal contraceptive method; CHOICE: Contraceptive Health Research of Informed Choice Experience; COCs: Combined oral contraceptives; ESC: European Society of Contraception and Reproductive Health; HCP: Health care professional.

## Competing interests

Merck, Sharp & Dohme (MSD) provided financial support for this study. The authors received no compensation or honoraria in association with this manuscript and exercised sole and complete editorial responsibility for this paper. BJO and MMP are employees of MSD and may potentially own stock and/or hold stock options in the company. CE has received payments for consulting and lecturing for MSD, Pfizer, and Sanova and has received research funding from MSD, a company that may have a commercial interest in the results of this study. BF and JB have received consulting fees and honoraria for lectures by MSD. SW has received consulting payments from MSD. SW has received consulting payments from MSD.

## Authors’ contributions

The authors of this manuscript all provided expert advice regarding the design and conduct of the CHOICE study and the interpretation and presentation of the data; they also contributed to the writing of this manuscript. In addition, JB led a team of representatives selected from the European Society of Contraception and Reproductive Health, a group that reviewed the study plan and provided input into the creation of a counseling leaflet that contained information about the pill, patch, and ring. BJO was the international project leader of the study, while MMP was responsible for statistical analysis. All authors read and approved the final manuscript.

The International Steering Committee of CHOICE included Dr. Christian Egarter (Austria), Dr. Steven Weyers (Belgium), Dr. Tomáš Fait (Czech Republic), Dr. Arie Yeshaya (Israel), Dr. Frans Roumen (the Netherlands), Prof. Leszek Pawelczyk (Poland), Dr. Marina Anatolyevna Tarasova (Russia), Prof. Vera Prilepskaya (Russia), Dr. Vladimir Cupanik (Slovakia), Prof. Kristina Gemzell-Danielsson (Sweden), Prof. Johannes Bitzer (Switzerland), Dr. Brigitte Frey Tirri (Switzerland), and Dr. Vyacheslav Kaminskiy (Ukraine).

## Pre-publication history

The pre-publication history for this paper can be accessed here:

http://www.biomedcentral.com/1472-6874/13/9/prepub

## Supplementary Material

Additional file 1: Table S1 Four most frequently cited reasons women selected the pill, patch or ring after counseling (all countries combined) ^†^. Click here for file

Additional file 2: Table S2 Four most frequently cited reasons women did not select the pill, patch or ring (all countries combined) ^†^. Click here for file
