# Supplementary material for: Women’s perceptions and reasons for choosing the pill, patch, or ring in the CHOICE study: a cross-sectional survey of contraceptive method selection after counseling
Source: BMC Womens Health. 2013 Feb 28;13:9. doi: 10.1186/1472-6874-13-9 (PMC3605181; doi:10.1186/1472-6874-13-9)
Supplement: Additional file 1: Table S1 — Four most frequently cited reasons women selected the pill, patch or ring after counseling (all countries combined) †. [file 1472-6874-13-9-S1.doc]

**Supplemental Table 1.** Four most frequently cited reasons women selected the pill, patch or ring after counseling (all countries combined).^†^

|  | **Country** | | | | | | | | | | |
| --- | --- | --- | --- | --- | --- | --- | --- | --- | --- | --- | --- |
|  | **All**  **(N=18,787)** | **Austria**  **(N=2478)** | **Belgium**  **(N=1801)** | **Israel**  **(N=1802)** | **Netherl**  **(N=727)** | **Sweden**  **(N=1944)** | **Switzerl**  **(N=2629)** | **CZ&SK**  **(N=1954)** | **Poland**  **(N=1836)** | **Russia**  **(N=1749)** | **Ukraine**  **(N=1867)** |
| Top four reasons why women selected the pill: n (%) | | | | | | | | | | | |
| N (Non-missing) | 9418 | 1393 | 939 | 991 | 423 | 1069 | 1461 | 905 | 953 | 843 | 441 |
| Easy to use | 6351 (67.4) | 987  (70.9) | 573  (61.0) | 641  (64.7) | 194  (45.9) | 823  (77.0) | 1018 (69.7) | 595  (65.7) | 700  (73.5) | 564  (66.9) | 256  (58.0) |
| Regular menstrual bleeding | 6184 (65.7) | 868  (62.3) | 631  (67.2) | 629  (63.5) | 251  (59.3) | 707  (66.1) | 980  (67.1) | 544  (60.1) | 694  (72.8) | 612  (72.6) | 268  (60.8) |
| Relief from menstrual pain | 4556 (48.4) | 627  (45.0) | 351  (37.4) | 425  (42.9) | 154  (36.4) | 446  (41.7) | 720  (49.3) | 489  (54.0) | 554  (58.1) | 507  (60.1) | 283  (64.2) |
| Convenience | 4197 (44.6) | 262  (18.8) | 466  (49.6) | 541  (54.6) | 259  (61.2) | 679  (63.5) | 378  (25.9) | 422  (46.6) | 530  (55.6) | 476  (56.5) | 184  (41.7) |
| Top four reasons why women selected the patch: n (%) | | | | | | | | | | | |
| N (non-missing) | 1541 | 190 | 94 | 214 | 16 | 117 | 182 | 87 | 266 | 176 | 199 |
| Easy to use | 1165  (75.6) | 133  (70.0) | 62  (66.0) | 158  (73.8) | 5  (31.3) | 96  (82.1) | 141  (77.5) | 53  (60.9) | 219  (82.3) | 147  (83.5) | 151 (75.9) |
| Weekly use | 1053  (68.3) | 133  (70.0) | 55  (58.5) | 139  (65.0) | 9  (56.3) | 80 (68.4) | 118  (64.8) | 80  (92.0) | 240  (90.2) | 104  (59.1) | 95  (47.7) |
| Convenience | 1039  (67.4) | 52  (27.4) | 74  (78.7) | 149  (69.6) | 10  (62.5) | 90  (76.9) | 83  (45.6) | 58  (66.7) | 221  (83.1) | 141  (80.1) | 161  (80.9) |
| Will not forget it | 819  (53.1) | 77  (40.5) | 58  (61.7) | 121  (56.5) | 5  (31.3) | 76  (65.0) | 97  (53.3) | 52  (59.8) | 160  (60.2) | 84  (47.7) | 89  (44.7) |
| Top four reasons why women selected the ring: n (%) | | | | | | | | | | | |
| N (non-missing) | 5520 | 577 | 483 | 403 | 130 | 430 | 737 | 825 | 458 | 593 | 884 |
| Monthly use | 4128  (74.8) | 452  (78.3) | 319  (66.0) | 241  (59.8) | 84  (64.6) | 314  (73.0) | 560  (76.0) | 780  (94.5) | 432  (94.3) | 389  (65.6) | 557  (63.0) |
| Convenience | 3361  (60.9) | 190  (32.9) | 280  (58.0) | 227  (56.3) | 76  (58.5) | 257  (59.8) | 363  (49.3) | 481  (58.3) | 362  (79.0) | 451  (76.1) | 674  (76.2) |
| Recommended by my doctor | 3338  (60.5) | 263  (45.6) | 243  (50.3) | 279  (69.2) | 57  (43.8) | 194  (45.1) | 329  (44.6) | 625  (75.8) | 311  (67.9) | 427  (72.0) | 610  (69.0) |
| Steady, low hormone levels | 3246  (58.8) | 235  (40.7) | 235  (48.7) | 220  (54.6) | 70  (53.8) | 304  (70.7) | 438  (59.4) | 467  (56.6) | 276  (60.3) | 411  (69.3) | 590  (66.7) |

^†^The four most frequently cited reasons women selected the pill, patch, or ring are shown for all countries combined. Although results from individual countries are also shown, these four reasons were not necessarily the four most frequently cited reasons in a given country.

Country: all, all countries combined; Netherl, Netherlands; Switzerl, Switzerland; CZ&SK, Czech Republic and Slovakia.
