# Supplementary material for: Women’s perceptions and reasons for choosing the pill, patch, or ring in the CHOICE study: a cross-sectional survey of contraceptive method selection after counseling
Source: BMC Womens Health. 2013 Feb 28;13:9. doi: 10.1186/1472-6874-13-9 (PMC3605181; doi:10.1186/1472-6874-13-9)
Supplement: Additional file 2: Table S2 — Four most frequently cited reasons women did not select the pill, patch or ring (all countries combined) †. [file 1472-6874-13-9-S2.doc]

**Supplemental Table 2.** Four most frequently cited reasons women did not select the pill, patch or ring (all countries combined).^†^

| **(A)** | **Country** | | | | | | | | | | |
| --- | --- | --- | --- | --- | --- | --- | --- | --- | --- | --- | --- |
|  | **All** | **Austria** | **Belgium** | **Israel** | **Netherl** | **Sweden** | **Switzerl** | **CZ&SK** | **Poland** | **Russia** | **Ukraine** |
| Top four reasons why women who selected the weekly patch did not select the daily pill: n (%) | | | | | | | | | | | |
| N (non-missing) | 1541 | 190 | 94 | 214 | 16 | 117 | 182 | 87 | 266 | 176 | 199 |
| Daily use | 1078  (70.0) | 134  (70.5) | 58  (61.7) | 117  (54.7) | 8  (50.0) | 78  (66.7) | 122  (67.0) | 68  (78.2) | 202  (75.9) | 145  (82.4) | 146  (73.4) |
| Will forget to take it | 1011  (65.6) | 117  (61.6) | 69  (73.4) | 126  (58.9) | 9  (56.3) | 84  (71.8) | 108  (59.3) | 56  (64.4) | 192  (72.2) | 115  (65.3) | 135  (67.8) |
| More convenient methods available | 721  (46.8) | 34  (17.9) | 41  (43.6) | 63  (29.4) | 2  (12.5) | 66  (56.4) | 68  (37.4) | 40  (46.0) | 155  (58.3) | 110  (62.5) | 142  (71.4) |
| Efficacy reduced by vomiting, diarrhea | 664  (43.1) | 120  (63.2) | 54  (57.4) | 52  (24.3) | 6  (37.5) | 35  (29.9) | 77  (42.3) | 43  (49.4) | 134  (50.4) | 67  (38.1) | 76  (38.2) |
| Top four reasons why women who selected the monthly ring did not select the daily pill: n (%) | | | | | | | | | | | |
| N (non-missing) | 5520 | 577 | 483 | 403 | 130 | 430 | 737 | 825 | 458 | 593 | 884 |
| Daily use | 4005  (72.6) | 418  (72.4) | 277  (57.3) | 221  (54.8) | 67  (51.5) | 295  (68.6) | 545  (73.9) | 675  (81.8) | 380  (83.0) | 495  (83.5) | 632  (71.5) |
| Will forget to take it | 3777  (68.4) | 377  (65.3) | 316  (65.4) | 242  (60.0) | 81  (62.3) | 271  (63.0) | 538  (73.0) | 561  (68.0) | 319  (69.7) | 424  (71.5) | 648  (73.3) |
| More convenient methods are available | 2643  (47.9) | 155  (26.9) | 154  (31.9) | 95  (23.6) | 33  (25.4) | 218  (50.7) | 288  (39.1) | 443  (53.7) | 242  (52.8) | 383  (64.6) | 632  (71.5) |
| Efficacy reduced by vomiting, diarrhea | 2503  (45.3) | 347  (60.1) | 214  (44.3) | 97  (24.1) | 30  (23.1) | 164  (38.1) | 365  (49.5) | 386  (46.8) | 255  (55.7) | 238  (40.1) | 407  (46.0) |
| Top four reasons why women who selected the daily pill did not select the weekly patch: n (%) | | | | | | | | | | | |
| N (non-missing) | 9418 | 1393 | 939 | 991 | 423 | 1069 | 1461 | 905 | 953 | 843 | 441 |
| Not discrete, visible | 4727  (50.2) | 752  (54.0) | 398  (42.4) | 430  (43.4) | 97  (22.9) | 583  (54.5) | 714  (48.9) | 467  (51.6) | 616  (64.6) | 415  (49.2) | 255  (57.8) |
| Can fall off | 4292  (45.6) | 667  (47.9) | 291  (31.0) | 438  (44.2) | 91  (21.5) | 481  (45.0) | 639  (43.7) | 391  (43.2) | 568  (59.6) | 541  (64.2) | 185  (42.0) |
| Can irritate skin | 3228  (34.3) | 473  (34.0) | 259  (27.6) | 296  (29.9) | 91  (21.5) | 334  (31.2) | 408  (27.9) | 349  (38.6) | 393  (41.2) | 444  (52.7) | 181  (41.0) |
| More convenient methods are available | 3115  (33.1) | 405  (29.1) | 274  (29.2) | 347  (35.0) | 127 (30.0) | 449  (42.0) | 397  (27.2) | 207  (22.9) | 347  (36.4) | 390  (46.3) | 172  (39.0) |

**[Supplemental Table 2 continued]**

| **(B)** | **Country** | | | | | | | | | | | |
| --- | --- | --- | --- | --- | --- | --- | --- | --- | --- | --- | --- | --- |
|  | **All** | **Austria** | **Belgium** | **Israel** | **Netherl** | **Sweden** | **Switzerl** | **CZ&SK** | **Poland** | **Russia** | **Ukraine** | |
| Top four reasons why women who selected the monthly ring did not select the weekly patch: n (%) | | | | | | | | | | | | |
| N (non-missing) | 5519 | 577 | 483 | 403 | 130 | 430 | 736 | 825 | 458 | 593 | 884 | |
| Not discrete, visible | 3184 (57.7) | 316 (54.8) | 215 (44.5) | 217 (53.8) | 35  (26.9) | 241  (56.0) | 413 (56.1) | 491 (59.5) | 328 (71.6) | 299 (50.4) | 629  (71.2) | |
| Can fall off | 2936 (53.2) | 286 (49.6) | 186 (38.5) | 205 (50.9) | 38  (29.2) | 238  (55.3) | 352 (47.8) | 466 (56.5) | 297 (64.8) | 393 (66.3) | 475  (53.7) | |
| More convenient methods are available | 2778 (50.3) | 201 (34.8) | 207 (42.9) | 166 (41.2) | 44  (33.8) | 246  (57.2) | 321 (43.6) | 374 (45.3) | 236 (51.5) | 367 (61.9) | 616  (69.7) | |
| Can irritate skin | 2589 (46.9) | 236 (40.9) | 150 (31.1) | 157 (39.0) | 26  (20.0) | 186  (43.3) | 286 (38.9) | 477 (57.8) | 249 (54.4) | 338 (57.0) | 484  (54.8) | |
| Top four reasons why women who selected the daily pill did not select the monthly ring: n (%) | | | | | | | | | | | |  |
| N (non-missing) | 9418 | 1393 | 939 | 991 | 423 | 1069 | 1461 | 905 | 953 | 843 | 441 | |
| Don’t like to use foreign body | 4427 (47.0) | 692(49.7) | 342(36.4) | 523(52.8) | 155(36.6) | 379(35.5) | 589(40.3) | 420(46.4) | 498(52.3) | 573(68.0) | 256(58.0) | |
| More convenient methods are available | 3628 (38.5) | 554(39.8) | 325(34.6) | 361(36.4) | 148(35.0) | 537(50.2) | 482(33.0) | 260(28.7) | 411(43.1) | 386(45.8) | 164(37.2) | |
| Not comfortable inserting ring in vagina | 3537(37.6) | 505(36.3) | 305(32.5) | 400(40.4) | 96(22.7) | 443(41.4) | 567(38.8) | 286(31.6) | 400(42.0) | 347(41.2) | 188(42.6) | |
| Don’t know anybody who uses it | 3256(34.6) | 351(25.2) | 229(24.4) | 482(48.6) | 174(41.1) | 428(40.0) | 492(33.7) | 288(31.8) | 398(41.8) | 261(31.0) | 153(34.7) | |
| Top four reasons why women who selected the weekly patch did not select the monthly ring: n (%) | | | | | | | | | | | | |
| N (non-missing) | 1541 | 190 | 94 | 214 | 16 | 117 | 182 | 87 | 266 | 176 | 199 | |
| Don’t like to use foreign body | 881(57.2) | 102(53.7) | 44 (46.8) | 139(65.0) | 8 (50.0) | 47 (40.2) | 81 (44.5) | 51 (58.6) | 155(58.3) | 130(73.9) | 124 (62.3) | |
| Not comfortable inserting ring in vagina | 784 (50.9) | 93 (48.9) | 57 (60.6) | 122(57.0) | 8 (50.0) | 60 (51.3) | 100(54.9) | 45 (51.7) | 117(44.0) | 83(47.2) | 99 (49.7) | |
| More convenient methods are available | 778 (50.5) | 76 (40.0) | 45 (47.9) | 105(49.1) | 7 (43.8) | 68 (58.1) | 80 (44.0) | 36 (41.4) | 137(51.5) | 105(59.7) | 119 (59.8) | |
| Not easy to use | 538 (34.9) | 53 (27.9) | 47 (50.0) | 67 (31.3) | 5 (31.3) | 37 (31.6) | 58 (31.9) | 24 (27.6) | 103(38.7) | 66 (37.5) | 78 (39.2) | |

^†^The four most frequently cited reasons women did not select the pill, patch, or ring are shown for all countries combined. Although results from individual countries are also shown, these four reasons were not necessarily the four most frequently cited reasons in a particular country. Country: All, all countries combined; Netherl, Netherlands; Switzerl, Switzerland; CZ&SK, Czech Republic and Slovakia.
